# Impact of oral microbiota on pathophysiology of GVHD

**DOI:** 10.3389/fimmu.2023.1132983

**Published:** 2023-03-09

**Authors:** Akira Yamamoto, Yui Kambara, Hideaki Fujiwara

**Affiliations:** ^1^ Department of Hematology and Oncology, Okayama University Hospital, Okayama, Japan; ^2^ Department of Hematology and Oncology and Respiratory Medicine, Okayama University Graduate School of Medicine, Dentistry and Pharmaceutical Sciences, Okayama, Japan

**Keywords:** oral microbiota, GvHD, dysbiosis, allogeneic transplantation of hematopoietic cells, prediction, HSCT

## Abstract

Allogeneic transplantation of hematopoietic cells is the only curative therapy for several hematopoietic disease in which patients receive cytotoxic conditioning regimens followed by infusion of hematopoietic stem cells. Although the outcomes have improved over the past decades, graft-versus-host-disease (GVHD), the most common life-threatening complication, remains a major cause of non-relapse morbidity and mortality. Pathophysiology of acute GVHD characterized by host antigen-presenting cells after tissue damage and donor T-cells is well studied, and additionally the importance of recipient microbiota in the intestine is elucidated in the GVHD setting. Oral microbiota is the second most abundant bacterial flora in the body after the intestinal tract, and it is related to chronic inflammation and carcinogenesis. Recently, composition of the oral microbiome in GVHD related to transplantation has been characterized and several common patterns, dysbiosis and enrichment of the specific bacterial groups, have been reported. This review focuses on the role of the oral microbiota in the context of GVHD.

## Introduction

Hematopoietic stem cell transplantation (HSCT) is a curative therapy for refractory diseases of the hematopoietic system. Graft-versus-host-disease (GVHD), a major adverse effect of allogeneic HSCT, was first observed in a mouse HSCT model in 1956. The model demonstrated the clinical manifestations of acute GVHD (aGVHD), such as liver damage, skin rash, and diarrhea (1). Continued research on aGVHD has revealed that its immune cell mechanisms mainly involve donor lymphocytes and host antigen presenting cells (APC)s, and that human leukocyte antigen (HLA) is important for the activation of allogeneic T cells (2). Acute GVHD is traditionally described in the following steps. First, the activation of host APCs associated with the conditioning regimen occurs, followed by the activation of donor T cells and target tissue damage due to cellular and inflammatory factors (3). Conditioning regimens based on high-dose chemotherapy and total body irradiation are important to eradicate the hematopoietic disease and immune cells in recipients that cause graft rejection and can lead to systemic tissue damage. Host tissues release inflammatory cytokines such as interleukin (IL)-1, IL-6, and tumor necrosis factor, which increase the expression of major histocompatibility complex antigens and cell surface adhesion molecules on host cells. Tissue damage, including that in the intestinal tracts, lead to the release of damage-associated molecular patterns (DAMPs) from host tissues and pathogen-associated molecular patterns (PAMPs) from the bacterial flora, resulting in a constant and excessive inflammatory and pro-inflammatory response (4). This cytokine storm activates host APCs, which present allogeneic antigens that are recognized by donor T cells, leading to T-cell activation (5). Second, activated T-cells proliferate and differentiate; CD4^+^ T cells differentiate into various subsets, including Th1, Th2, Th9, Th17, and Th22, and CD8^+^ T cells differentiate into cytotoxic T cells, which migrate and cause tissue damage in target organs such as liver, skin, and intestine to attack the host (6). Donor T cells are important targets for immunosuppressive therapy in the prevention and treatment of aGVHD, including anti-thymocyte globulin, calcineurin inhibitors, mToR inhibitors, mycophenolic acid, methotrexate, post-transplant cyclophosphamide, and corticosteroids (4, 7–9). HLA is the major host protein recognized by donor T cells (10). HLA class I (A, B, C) is present in almost all nucleated cells in the body, while class II (DR, DQ, DP) is mainly expressed in hematopoietic stem cells and its expression can be induced in other cells by inflammation. The incidence of aGVHD is related to the degree of HLA mismatch, and all HLA of the donor and recipient should be identical. About half of allogeneic HSCT patients develop grade II to IV aGVHD, and most patients respond to corticosteroids treatment. Approximately 40% of patients who receive HLA identical grafts develop aGVHD, which requires treatment with corticosteroids. Some patients experience steroid-refractory aGVHD with poor overall survival, ranging from 5–30% (11). This has been attributed to differences in gene-encoding proteins called minor histocompatibility antigens, which are located outside the HLA locus (10).

Owing to these limitations of immunosuppressive therapy and HLA-matched prophylaxis in the prevention and treatment of aGVHD-targeting immune-cells, a third pathophysiological target has focused on host tissue factors in the past decade (12, 13). One of the most extensively studied areas is the relationship between intestinal microbiota and GVHD. The intestinal tract, injured by diverse factors such as preconditioning regimens including Total Body Irradiation (TBI) and high-dose chemotherapy, infection, decreased oral intake, antibiotics, and immunosuppressive therapy, is a primary aGVHD target organ and plays a major role in the pathogenesis of aGVHD (14). Approximately 100 trillion prokaryotic cells inhabit the intestine, most of which are biochemically anaerobic bacteria. Three major phyla of these bacteria, *Bacteroidetes*, *Firmicutes*, and *Actinobacteria*, comprise over 90% of the community (15). Next-generation sequencing has revealed that the gut microbiota harbors 1,000–1,150 bacterial species at the population level, with each individual harboring at least 160 species (16). The mammalian gastrointestinal tract is a relatively hypoxic tissue. Aerobic and facultative anaerobic bacteria consume oxygen in the distal intestinal tract, keeping the lumen hypoxic, colonized by anaerobic bacteria that produce short-chain fatty acids (SCFAs) (17). Intestinal epithelial cells are adapted to this hypoxic environment and play a central role in the homeostasis of the entire intestinal tract, including the intestinal microbiota. Intestinal epithelial cells are continuously renewed from intestinal stem cells (18). Energy production involves β-oxidation and oxidative phosphorylation of fatty acids by mitochondria, which consume large amounts of oxygen and reduce oxygen pressure in the lumen (19). This oxygen consumption helps anchor the polarized anaerobic bacteria that produce carbohydrate metabolites (20). Epithelial metabolism of SCFA is an important factor in physiological hypoxia of the mucosa with stabilization of oxygen consumption and hypoxia-inducible factors that promote intestinal protection (21).

The gut microbiota is significantly less diverse during the transplant period. Profiling of 8,767 fecal samples from 1,362 patients at four centers showed a lower risk of mortality with higher diversity of the gut microbiota. A subgroup analysis showed that lower diversity was associated with a higher risk of transplant-related mortality and mortality attributable to aGVHD. Furthermore, groups with low diversity in stool samples before transplantation had a lower survival rate (22). In a retrospective analysis of 80 patients, the group with lower diversity of microbiota in feces at the time of neutrophil engraftment had a significantly increased mortality rate compared to the group with higher diversity. This was also evident in a multivariate analysis that included other clinical predictors and showed that microbiota diversity was an independent predictor (23). Decreased diversity of the intestinal microbiota is associated with prechemotherapy, preconditioning regimens, drugs including antibiotics and immunosuppressant, loss of appetite, and injury to the gastrointestinal epithelium attributable to these therapies (24, 25). Studies have also examined the association between allogeneic HSCT and specific bacteria. Fecal samples from 38 transplant patients at a single institution at five time points (preconditioning, 1, 3, 6, and 12 months post-transplant) analyzed using 16S rRNA analysis showed that an increase in the abundance of *Enterococcus* spp. in the gut at 1 month post-transplant was associated with a decreased overall survival rate, suggesting a promising prognostic indicator (26). Additionally, a higher bacterial population composed of *Eubacterium limosum* in the gut in the early post-transplant period has been associated with a lower the risk of disease recurrence and progression, based on the analysis of data from a single institution (27). It has been observed that an increase in the abundance of *Enterococcus* is associated with aGVHD and mortality. The increase in the abundance of *Enterococcus* was dependent on the disaccharide lactose, and dietary lactose deficiency suppressed *Enterococcus* abundance, followed by reduced aGVHD severity in a gnotobiotic mouse model (28). In patients undergoing allogeneic HSCT, the oral-origin genera *Rothia*, *Solobacterium*, and *Veillonella* were identified in the stools, and they were positively correlated with aGVHD (29). The use of multiple broad-spectrum antibiotics is often unavoidable during allogeneic HSCT. Undesirable consequences of antibiotic use in cancer patients have been observed, including antibiotic resistance, predominance of pathogenic bacteria, transient or profound loss of microbial diversity, increased susceptibility to infection, and risk of recurrent infections (30). While aztreonam and cefepime have not been associated with aGVHD-related mortality, tazobactam and piperacillin have been shown to have a profound effect on intestinal dysbiosis after allogeneic HSCT. Imipenem and cilastatin treatment in aGVHD mouse model was shown to decrease the protective intestinal mucus and intestinal barrier function with an increase of *Akkermansia muciniphia*, an indigenous bacterium with mucolytic capacity (31). In a clinical study that evaluated the effects of prophylactic and therapeutic antibiotic administration prior to day 0 of allogeneic HSCT, the antibiotic group had a significantly higher incidence of aGVHD and shorter survival (32). Early antibiotic exposure in allogeneic HSCT was associated with lower urinary 3-indoxyl sulfate levels, lower fecal indigenous clostridia, and higher transplant-related mortality (33). While negative outcomes of broad-spectrum antibiotic administration are available, some studies have reported the contrary. In mice with diet-induced obesity, pre-transplant prophylactic antibiotic treatment had a protective effect against aGVHD in terms of production of endotoxin and inflammatory cytokines, pathological changes in the intestinal tract, and mortality. Some controversial aspects regarding antibiotics and aGVHD remain (34). Although prophylactic and systemic antibiotics clearly improve transplant-related mortality, especially infection-related mortality, comprehensive decisions regarding antibiotic use around the time of HSCT may need to be made, taking into account disease status, donor source, history of antibiotic use, microbial injury status, and other risk factors (12). In chronic GVHD, plasma levels of SCFAs propionate and butyrate were found to be low in patients with chronic GVHD. This finding suggests that the SCFA levels are at least partially involved in systemic immune regulatory functions due to reduced gut bacterial function (35). Butyrate is fermented by anaerobic bacteria in the colon and has a local protective effect by serving as an energy source for colonocytes, suppressing oxidative stress, and inducing T cell differentiation into Tregs *via* histone acetylation (36, 37). These changes in anaerobic bacteria are affected by antibiotics and environmental changes associated with tissue metabolic changes (38). Quinolones, commonly used for prophylaxis against febrile neutropenia in patients undergoing transplantation, are broad-spectrum bactericides effective against many gram-positive and gram-negative bacteria, targeting the bacterial enzymes DNA gyrase and DNA topoisomerase, which are essential for DNA replication and repair. When antibiotics were administered to healthy participants, a single dose of clindamycin, a lincosamide, and ciprofloxacin, a quinolone, had a negative effect on butyric acid-producing bacteria in the gut for several months and had a major effect on SCFA production (39). Butyrate is reduced during the transplantation period, followed by decreased histone acetylation (40). These changes result in a loss of intestinal epithelial integrity, which promotes bacterial lipopolysaccharide exudation and donor-reactive T cell activation (41). Subsequently, through the activation of caspase-11, a receptor for LPS, and cleavage of gasdermin D, IL-1α was released and it exacerbated aGVHD in a mouse model (42). Therapeutic interventions targeting the gut microbiota currently include the various approaches (41). Limited exposure to antibiotics that eliminate mainly anaerobic commensal bacteria may be considered. However, in many cases where broad-spectrum antibiotics are required to prevent or treat severe infections, including febrile neutropenia, this is difficult to implement. Therefore, the proper use of antibiotics is encouraged. Open and two-armed randomized controlled nutrition intervention trials in patients with hematologic malignancies undergoing allogeneic HSCT, have examined associations among gut microbiota, SCFAs, markers of gut barrier function, and clinical outcomes; however, no differences were observed between the intervention and control groups (43). Administration of meropenem, a frequently used broad-spectrum antibiotic, caused thinning of the intestinal mucous layer *via* an increase in the abundance of *Bacteroides thetaiotaomicron*, followed by the exacerbation of aGVHD in mice. Interestingly, administration of xylose, the level which was reduced in the colonic lumen during allogeneic HSCT, prevented thinning of the mucus layer of the colon (44). As another strategy that targets gut microbiota, fecal microbiota transplantation (FMT) is currently being developed, and several clinical trials have already been conducted. In the most recent meta-analysis of 242 patients with steroid-dependent GVHD, 100 patients achieved a complete response and 61 achieved a partial response to FMT. Although 2% of patients had FMT-related infections, all patients recovered after treatment. Other adverse effects were mild, and FMT shows a promise as the new treatment strategy for GVHD (45).

The oral microbiota, the second most abundant bacterial community in the human gut, has recently attracted attention with comprehensive research on intestinal microbiota. The oral microbiota serves as a reservoir of the intestinal microbiota, and they are jointly involved in systemic disease development (46, 47). The association between oral bacteria and systemic diseases was first proposed in 1891 (48). In recent years, development of new analytical methods such as next-generation sequencing has led to comprehensive analyses and research on non-culturable bacteria, which had been challenging to perform in the past (49). Until recently, the direct transfer of oral bacteria to the intestinal tract was considered extremely rare, at least in healthy individuals. Recently, it has become clear that oral bacteria can migrate to the intestinal tract even in healthy individuals (50). In certain diseases, oral bacteria migrate directly through the gastrointestinal tract and establish there. In addition to direct transfer, inflammation in the oral cavity such as periodontal disease may trigger systemic inflammation and systemic diseases. The oral microbiota is closely related to the intestinal microbiota, and the possible existence of a “mouth–gut axis” has been demonstrated in some diseases such as inflammatory bowel disease (IBD) (51). In this review, we focus on the relationship between the two communities of microbiota in relation to HSCT and the adverse effects, reviewing the limited literature on oral microbiota and HSCT.

## Oral microbiota

In 2007, the human microbiome project analyzed the baseline microbial and functional diversity of the human microbiota and established 48 major microbiomes in the human body (52). The oral microbiota is the second largest, after the intestinal microbiota (53). Immunotolerance to the oral microbiota begins before birth. Microorganisms are present in the placenta, amniotic fluid, and umbilical cord blood (54). Furthermore, maternal oral bacteria access the placenta through the gingiva, and fetal regulatory T cells prevent this undesirable homologous reactivity. As a result, the fetus acquires prenatal tolerance to the maternal oral bacteria, which also affects the development of the immune system after birth (55). Oral bacteria colonize rapidly after birth, establishing microbiota at each site in cooperation with the host’s immune system (55). Infants born *via* vaginal delivery show high diversity in oral bacteria in the first 3 months of life (56). The microbiota differs by the mode of delivery. *Streptococcus mutans*, a causative microorganism of dental caries, is acquired in infants born *via* cesarean section approximately 1 year earlier than those born *via* vaginal delivery (57). The oral cavity is considered a challenging environment for the microbes to survive after birth, due to the daily fluctuations in nutritional supply, temperature, pH, mechanical forces of chewing, and hygiene practices, and exposure to chemicals from hygiene, pharmaceuticals, toxic substances, and smoking products. However, a study that followed healthy individuals for 7 years found that the oral microbiota was stable over a long time and that these factors may cause transient changes (58).

The human oral cavity contains different habitats, including teeth, gingival sulcus, tongue, buccal mucosa, hard palate, soft palate, and tonsils, which are colonized by a wide variety of bacteria, with about 1000 species at the species level and different subsets predominating each habitat (49, 59). These surfaces are exposed to saliva and gingival crevicular fluid in the subgingival margin. Primary colony formers in the oral cavity are predominantly facultative anaerobes such as streptococci and actinomycetes. *Streptococcus* accounts for over 80% of the initial components of biofilms. Most of them are commensal and classified into five groups: *mutans*, *salivarius*, *anginosus*, *sanguinis*, and *mitis* (60). Of these, *S. mutans* has been studied extensively as a caries-causing organism in terms of the ability to metabolize sugars and produce large amounts of extracellular polymers from sucrose, and resistance to environmental stress. It has also been implicated in endocarditis, IgA nephropathy, and atherosclerosis (61). In the subgingival region, the abundance of anaerobic bacteria such as *Bacteroidaceae* and *Spirochaetes* increases due to decreased oxygen levels (62). The oral cavity has multiple commensal environments, each with different microbiota, but the microbiomes of the tongue and saliva are consistent (63, 64). Oral bacteria have been well studied because of ease of collection; however, more than half of these oral microbiota are unculturable bacteria. The use of new analytical methods, such as next-generation sequencing, has revealed their complex structures (49). Most oral microbiota exists in the form of oral biofilms, except in the saliva (65). Biofilms have been studied for their role since Costerton et al. published the mechanism of bacterial adhesion in the oral cavity in 1978 (66). A biofilm is defined as an aggregate of bacterial cells adhering to an inert surface or biological surface, surrounded by a self-produced extracellular polymeric matrix. Oral bacterial biofilms enable bacterial colonies to adapt to high cell densities; they are composed of proteins, lipids, polysaccharides, and extracellular DNA. Biofilms are microenvironments capable of regulating pH, oxygen level, and redox status in the oral cavity, improving nutrient availability for bacteria and protecting them against environmental stress (67). In addition to bacteria, viruses, fungi, and yeasts are endemic to the oral cavity. Information on these oral biomes is limited compared with that on the bacterial microbiota. The most common viral families present in healthy individuals include *Anaelloviridae*, *Papillomaviridae*, and *Herpesviride* (68). A recent study on fungi using sequencing of the taxonomically informative pan-fungal internally transcribed spacer gene with DNA extracted from oral rinses revealed 154 fungal species (69). *Candida* and *Aspergillus* were isolated from 100% of the participants, *Penicillium* in 97% of the participants, *Schizophyllum* in 93% of the participants, *Rhodotorula* in 90% of the participants, and *Gibberella* 83% of the participants. A trend toward an increase in the abundance of *Candida*, which is associated with periodontal disease, has been observed in individuals with periodontal disease; this trend is also observed with an increase in the number of permanent teeth lost (69). In addition, *Candida albicans* interacts with *Porphyromonas gingivalis*, known as one of the most prominent causative bacteria for periodontal disease, to exacerbate the virulence of *P. gingivalis*. A comprehensive study of the oral biome, not limited to bacteria, is required (70).

## Oral dysbiosis

**Figure 1:**
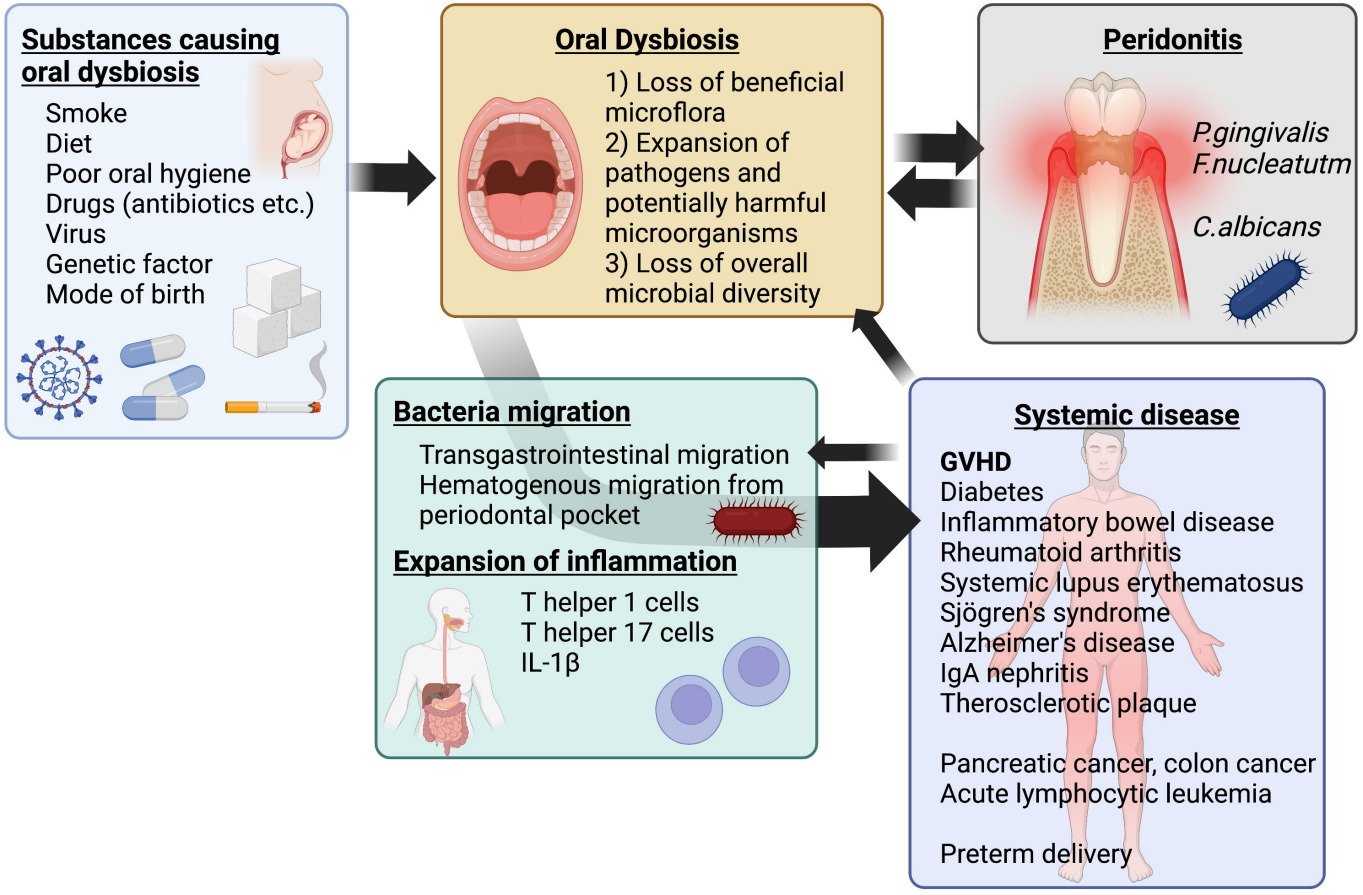
Schematic of oral dysbiosis. Oral dysbiosis is affected by environmental and genetic factors. In addition, it develops as a complementary exacerbating factor for periodontal disease and some systemic diseases, such as diabetes mellitus. The resulting systemic transfer of bacteria and spillover of inflammation contribute to systemic diseases, including GVHD after transplantation. Created with BioRender.com.

The oral microbiome contains various bacteria that act as a protective barrier against the establishment of pathogenic bacteria. Oral dysbiosis can lead to the establishment of pathogenic bacteria in the oral cavity (71). The saliva contains antimicrobial peptides and a host glycoprotein component that provides nutrition to oral bacteria, regulating the balance between protection and attack against bacteria (72). This fraction in the saliva varies considerably among individuals with age, health status, and disease state. For example, patients with type 1 diabetes mellitus have reduced salivary gland function, resulting in decreased salivary flow and decreased secretion of antibacterial enzymes such as lysozyme (73). Dysbiosis involves disruption of microbiome homeostasis caused by imbalance of microbiota, changes in the functional composition and metabolic activity of microbiota, and changes in local distribution of microflora. Dysbiosis is a combination of the following three conditions: 1) loss of beneficial microbiota, 2) expansion of pathogens and potentially harmful microorganisms, and 3) loss of overall microbial diversity (74). It is not limited to local effects, but can also promote specific systemic diseases in the host (65) (**Figure 1**).

Common causes of oral dysbiosis include diet, smoking, oral hygiene practices, and antibiotics (75). In the diet, the intake of sugars and certain foods such as farmed animal meat, dairy products, refined vegetable oils, and processed grains affects the composition of the microbiota. Although the oral microbiota is not dependent on diet as an energy source (76), persistent sugar intake results in dysbiosis associated with a shift to glycolytic and acidophilic microbiota (77). Regarding smoking, the oral microbiota of smokers differs from that of nonsmokers in many respects, including reduced diversity, bacterial abundance, and metabolites. In addition, these effects persist for several years after smoking cessation (78). Bacteria in oral biofilms are more resistant to antibiotics than planktonic bacteria in the saliva (79). Horizontal transmission of antibiotic resistance genes in oral biofilms is observed even in healthy individuals who have not been exposed to antibiotics (80). Interestingly, the resistance of microbiota in the saliva is more robust and stable than that of microbiota in the intestinal tract (39).

Periodontal disease is a disease that is typically associated with oral microbiota dysbiosis (51); it encompasses a wide range of chronic inflammatory conditions of the gingiva, bone, and tooth-supporting ligaments (81). It begins with localized gingivitis caused by bacteria in biofilms formed on the teeth and gingiva. Periodontal disease has multiple risk factors such as genetic factors, smoking, plaque accumulation, diabetes, and socioeconomic status, as reported by studies in the USA (81, 82). Diabetes mellitus has a reciprocal risk relationship with periodontal disease, as discussed below. Here, periodontitis occurs in a state of dysbiosis in which some pathogenic bacteria are relatively more abundant (83). Pathogenic bacteria that cause periodontitis are represented by *P. gingivalis* and *Fusobacterium nucleatum* (84). *Porphyromonas gingivalis* invades the epithelium and penetrates deep into tissues, evading humoral immune responses owing to its intracellular localization. Furthermore, it can degrade cell signaling molecules and inactivate tissue regeneration and homeostatic functions (85). As gingivitis progresses, gingiva, bone, and ligaments are damaged, leading to the formation of periodontal pockets, eventually causing systemic inflammation. Mouse models of periodontal disease can be classified as follows: the ligature model, in which periodontal tissue is rapidly destroyed locally by threading the teeth (86); the injection model, in which bacteria are microinjected directly into the periodontal lesions at the gingival margin (87); the mono-infection model, in which *P. gingivalis* is injected daily after oral administration of antibiotics mixed with vehicle (88); and the multi-infection model, in which *P. gingivalis*, *Treponema denticola*, *Tannerella gingivalis*, and *F. nucleatum* mixed with vehicle and injected daily (89). Recent research has included not only local studies in the oral cavity, but also those on the relationship with systemic diseases. When investigating causative factors of dysbiosis other than periodontal disease, the oral and intestinal microbiome in patients with COVID-19 was shown to have lost richness and evenness compared to those in the healthy group, and these findings revealed that certain viral infections can also cause oral dysbiosis (90, 91).

## Association between oral microbiota and systemic diseases

Periodontitis locally damages connective tissue and bone, followed by extensive inflammatory cell infiltration of periodontal pockets and connective tissue near the epithelium (92). The hypothesis that oral bacteria may be associated with systemic diseases was first proposed by Miller in 1891 (48). This was followed by the speculation that oral infections caused systemic diseases such as rheumatoid arthritis (93). The mechanism was thought to involve the entry of dental plaque and its metabolites into the blood circulation. Subsequently, a strong link between systemic immunity and the gut microbiota (94), and the association of the oral microbiome with many systemic diseases, including rheumatoid arthritis, Alzheimer’s disease, cardiovascular disease, diabetes, inflammatory bowel disease, premature birth, and cancer, were also demonstrated (95). All these diseases are linked to oral microbiota dysbiosis. A paradigm shift in microorganisms and cancer occurred with the discovery of the association between *Helicobacter pylori* and gastric cancer and lymphoma (96, 97). Since then, the association between bacteria and cancer has been widely studied, and it has become clear that cancer is also associated with oral microbiota.

The association between *P. gingivalis* and cancer has been widely studied, and *P. gingivalis* is strongly associated with the development and resistance to treatment of oral, esophageal, and pancreatic cancers (97–99). Oral gastrointestinal cancer-related mortality has been found to be associated with *P. gingivalis* antibody levels independently of periodontal disease (100). *Porphyromonas gingivalis*-infected epithelial cells 1) exhibit anti-apoptotic properties and inhibit chemically induced apoptosis with activation of Jak1/Akt/Stat3 signaling that regulates the intrinsic mitochondrial apoptotic pathway (101), and 2) manipulate CDK activity and decrease p53 levels, a tumor suppressor factor (102). Through these mechanisms, *P. gingivalis* is thought to be associated with carcinogenesis. While intestinal dysbiosis is a risk factor for colorectal cancer and colorectal adenomas, there are a few reports of specific bacteria as etiologic agents. *Fusobacterium nucleatum* is present in the epithelium of colorectal adenoma and colorectal cancer cells; it is relatively abundant compared with that in healthy controls, and has been found to promote rectal cancer development through Toll-like receptor (TLR) 2, TLR4, and microRNA-21 (103, 104). It has also been found that *F. nucleatum* on the buccal mucosa side migrates to the site of rectal cancer impairing the efficacy of radiation and anticancer drug therapy and worsening prognosis in a mouse model (105). In addition, the composition of the supragingival plaque microbiota in children with acute lymphocytic leukemia is less diverse than in the healthy group: the abundance of members of the phylum Firmicutes, the class Bacilli, the order Lactobacillales, the families *Aerococcaceae* and *Carnobacteriaceae* and the genera *Abiotrophia* and *Granulicatella* were mainly associated with acute lymphocytic leukemia (106).

It has been demonstrated that local inflammation caused by periodontitis affects not only cancers but also systemic disease a century after Miller’s first hypothesis. Diabetes and periodontal disease are mutually aggravating (107); oral microbiota dysbiosis induces insulin resistance by affecting the body’s immune inflammation and oxidative stress, adversely affecting diabetes (95). Conversely, elevated glucose levels in the saliva and tissues in patients with diabetes result in oral microbiota dysbiosis, with a shift to glycolytic and acidophilic microbiota (76, 77). Patients with rheumatoid arthritis, systemic lupus erythematosus, and Sjögren’s syndrome also have reduced oral microbiota diversity; the individual candidate pathogens differ among studies (108, 109). Regarding the cardiovascular system, infectious endocarditis is often caused by oral microbiota, and an increased risk of atherosclerotic plaque formation was reported in 1993 in patients with periodontitis (110). More recently, 23 species of oral bacteria have been found in treated atherosclerotic plaques, which are thought to have migrated hematogenously (111). Alzheimer’s disease remains an incurable disease that affects 47 million people worldwide. This disease has also been linked to periodontal disease, and the severity of periodontal disease, small number of remaining teeth, and irregular tooth brushing habits are risk factors for Alzheimer’s disease; preventive effects are expected by improving these factors (112–114). IgA nephropathy is one of the main causes of chronic kidney disease. Dysbiosis in saliva microbiome has been identified in the patient group, with more *Neisseria* spp. than in the healthy group (115). An association with preterm delivery has also been noted (116). In the last trimester of pregnancy, periodontal disease and dental caries-related oral microbiota predominate. Among women in the immediate postpartum period, especially those with poor oral hygiene, multidrug-resistant colonies are upregulated and a decrease in oral bacterial diversity is observed compared with that in nonpregnant women, suggesting the importance of oral management from pregnancy to postpartum (117). The examination of the gut microbiota of patients with IBD has revealed many oral microbiota, suggesting a direct recent migration. In addition, periodontitis and the involvement of nonbacterial microorganisms, viruses, and fungi such as *Candida albicans* have also been found to have an effect on IBD (118).

## Translocation of oral microbiota into the intestinal tract

Previously, it was believed that the oral microbiota did not migrate to the intestinal tract. Humans ingest 1–1.5 L of saliva daily, and over 99% of the millions of bacteria in the saliva were believed to be killed by gastric acid (119) and antibacterial bile acids (120) while passing through the acidic environment from the stomach to the small intestine, where both communities were thought to be separated. Profiling analysis of saliva and fecal samples from healthy adults for microbial single nucleotide variants revealed that 77% of the 125 bacterial species that predominate both in the mouth and intestinal tract showed oral–fecal transmission, indicating that oral microbiota migrates to the intestinal tract not only in people with disease but also healthy individuals (50). Conditions that may accelerate the migration from oral cavity to the intestinal tract include 1) periodontal disease, 2) systemic conditions, and 3) external factors such as drugs.

1) In a previous study, fecal and salivary samples from patients with severe periodontal disease and healthy donors were collected for analysis using 16SrRNA; the results showed that saliva-derived microorganisms were present in the feces of the periodontal disease group. In addition, the saliva-derived microorganisms settled in the intestinal tract in the mouse model (121). In addition to the mechanism by which bacteria migrate directly to the intestinal tract, another pathway has also been identified in which oral inflammation spills over and leads to intestinal inflammation *via* Th17 cells (51). *Porphyromonas gingivalis* induces oral inflammation such as periodontitis, which indirectly leads to systemic inflammation, and also migrates directly to the intestinal tract, causing dysbiosis of the intestinal microbiota, followed by disruption of the intestinal barrier (122).

2) In patients with certain diseases, there is an increase in the abundance of certain oral bacteria in the gut. For example, in rheumatoid arthritis, a decrease in the abundance *Haemophilus* spp. and an increase in the abundance of *Lactobacillus salivarius* have been observed in feces, teeth, and saliva (123). Furthermore, it has been found that species commonly considered as opportunistic pathogens become predominant in the oral cavity of patients with rheumatoid arthritis and colorectal cancer, and that mouth-to-gut microbial transmission is more frequent in such patients than in healthy individuals (50, 124). Mouth-derived bacterial abundance also increases in the gut of individuals with IBD (125). *Klebsiella* strains derived from the saliva of patients with IBD settle in the intestinal tract when fed to gnotobiotic mice, strongly induce Th1 cells and intestinal inflammation (46). These two pathways of direct migration and systemic spread of local inflammation exacerbate the progression of IBD.

3) Long-term use of proton pump inhibitors reduces the diversity of the gut microbiota and causes an increase in the abundance of *Rothia* spp. in the oral microbiome. These microbiota changes may increase the transfer of oral microbiota to the intestinal tract and the risk of *Clostridium difficile* infection (126).

In addition to bacterial transgastrointestinal migration, hematogenous systemic dissemination of oral bacteria from periodontal pockets, including that to the intestinal tract, has been associated with systemic diseases, especially atherosclerosis in periodontal disease (127). The oral microbiota serves as an important reservoir for maintaining the internal stability of the intestinal microecosystem, whereas pathogenic oral bacteria sometimes migrate into the intestinal tract *via* the digestive tract or blood and mediate diseases such as IBD (46, 47).

## Oral microbiota and HSCT

Oral mucositis (OM) is the most prevalent intraoral complication during HSCT, occurring in approximately 70-86.8% of patients following HSCT, due to mucosal damage associated with high-dose chemotherapy regimens and/or TBI (128, 129). This leads to increased pain, malnutrition, and risk of infection, resulting in high-risk predisposition to GVHD. Pretreatment including TBI is associated with a high incidence of OM. Specifically, cyclophosphamide has a 100% induction rate of OM over grade 3 as a World Health Organization HSCT complication (130). Severe OM is associated with days of total parenteral nutrition and parenteral drug therapy, days of fever, incidence of serious infections, duration of hospital stay, and total number of hospitalizations (131). A study on the salivary microbiome and metabolome in 184 patients showed that they had oral dysbiosis, and that pre-transplant *Kingella* and *Atopobium* levels were associated with future severe OM. *Methylobacterium* spp. were predominantly enriched in patients with severe OM. The metabolite that showed changes was polyamine, which is produced by commensal bacteria in the gut and is essential for mucosal homeostasis, maintenance of the mucosal barrier, and recovery after damage. This finding indicates that the oral microbiome is involved in the development of OM during transplantation (132).

It is not uncommon for multidrug antibiotic therapy to be administered as prophylactic or febrile neutropenia therapy during transplantation. Although the oral microbiota is better preserved after general antibiotic therapy compared to the gut microbiota, *Staphylococcus* spp. and *Enterococcus* spp. were predominant following multidrug antibiotic therapy during allogeneic HSCT: beta-lactam and glycopeptide combination therapy. Ulcerative oral mucositis after allogeneic HSCT has been observed only in the combination of multi-antibacterial therapy (133). In the analysis of oral rinses in patients undergoing allogeneic HSCT, the OM-affected group showed a substantial decrease in bacterial microbiota compared to the non-affected group for up to 3 weeks after transplantation (134). When neutrophil count was included in the regression model, it was found to be a confounding variable, affecting both mucositis and bacterial diversity, while there was no direct effect on diversity. Traditionally, oral hygiene with mouthwash is used to prevent OM after HSCT. In addition, treatment with folinic acid is related to a reduced incidence of OM induced by MTX, whereas there was no relationship in the incidence of aGVHD (135).

Oral microbiota and intestinal microbiota also undergo dysbiosis after HSCT, and the relationship between the pattern of microbiota and the recurrence rate, incidence of adverse effects, including GVHD, has been studied in humans while the association between oral microbiota and HSCT in mouse models has not been verified to date. Dysbiosis was observed in the oral microbiome, from preconditioning to long after allogeneic transplantation, regardless of its location. A decrease in diversity was observed in the dorsal tongue swab at preconditioning (136) and also in the supragingival dental biofilm early after allogeneic transplantation from preconditioning (137). Analysis of buccal swabs of children showed a decrease in diversity at 1 month after allogeneic transplantation, followed by reconstitution at 1–3 months (138). In addition, in cases with severe aplastic anemia, brushing of the back of the tongue showed a decrease in diversity at pre-conditioning, neutrophil engraftment, and at 100 days after autologous transplantation. A comparison with the human microbiome project database revealed that approximately 35% of the bacterial identifiers were unique to this tongue dorsum sample. *Rothia mucilaginosa* and *Haemophilus parainfluenzae* were significant among participants undergoing HSCT (139). This bacterial specificity was also evident in a study comparing the tongue microbiota of 45 patients with that of 164 healthy individuals. At the time of allogeneic transplantation, 146 taxa were identified withing the bacterial microbiota, 34 of which did not match the bacteria predominantly found in the oral cavity in the database (140). These changes in the early post-transplant period may be due to a significant reduction in defense mechanisms due to the conditioning regimen and associated neutropenia, and they may also be related to OM, antibiotic use, and decreased oral intake. Previous studies have shown that these changes return to approximately the pre-conditioning phase within 1–3 months (134, 138).

The risk of aGVHD was higher when *Streptococcus* and *Corynebacterium* abundance in the supragingival dental biofilm was high during preconditioning (**Table 1**). Conversely, high *Veillonella* abundance was associated with a low risk of aGVHD. This may be because lactic acid metabolism by *Veillonella* contributes to the resilience of the oral microbiota against acidification (77). Furthermore, *Enterococcus faecalis* bloom during transplantation was associated with acute and severe aGVHD risks (137). In pediatric data, the composition of the buccal microbiota was associated with the development of aGVHD and with CD4^+^ T cell, Th17 cell, and B cell reconstitution (138). Unfortunately, no study has directly examined whether the oral microbiota affects the intestinal microbiota, leading to subsequent transplant-related complications. A study simultaneously analyzing the intestinal, oral, and nasal microbiota at the time of transplantation in pediatric cases showed that not only the intestinal microbiota but also the relative increase in the abundance of oral *Actinomycetaceae*, *Prevotellaceae*, and *Propionibacteriaceae* at preconditioning could be a predictor of the development of aGVHD of grade II or higher. Risk assessment using oral microbiota, which is easier to collect than feces, is useful for clinical application (138) While some reports suggest that an increase in pathological bacteria and a decrease in indigenous bacteria are associated with the incidence of GVHD, it has been reported that dysbiosis of the tongue microbiota is not associated with the risk of acute and chronic GVHD (136). Similarly, it has been reported that there was no difference between the microbiota in oral rinses at preconditioning and that at the onset of chronic oral GVHD, and the observed dysbiosis during HSCT might not have been associated with the onset of chronic oral GVHD (134). The relationship between oral microbiota and GVHD remains controversial and open to further study. In an analysis of the tongue microbiota at preconditioning, predominance of a single genus was associated with a higher risk of recurrence at 3 years, PFS, and 3-year OS after allogeneic HSCT; decreased *Solobacterium* abundance was associated with an increased risk of recurrence (136). In the tongue microbiota at the time of transplantation, *Staphylococcus haemolyticus* and/or *Ralstonia pickettii* were significantly associated with a higher risk of death (140). Regarding the other complications during allogeneic HSCT, it has been reported that *Campylobacter rectus* and/or *Campylobacter concisus* were predominant in patients with respiratory complications within 100 days after HSCT, suggesting an association between oral microbiota and post-transplant respiratory complications (141).

**Table 1:** Human oral microbiota and HSCT.

Study	Year	Type of HSCT	Follow-up in months (median)	Increased strain	Sample collection site	Sample collection period	Characteristics
Ames et al. (141)	2012	Allo-HSCT (Adults)	100 days	*Campylobacter rectus* and *Campylobacter concisus*	Saliva, tongue, buccal surfaces and supragingival plaque	Before the conditioning regimen, at aplasia and at engraftment	Association between respiratory complications by 100 days after HSCT
Oku et al. (140)	2020	Allo-HSCT (adults)	9-42 (23)	*Staphylococcus haemolyticus, Ralstonia pickettii*	Tongue	The day of transplantation	Higher risk of mortality
Heidrich et al. (137)	2021	Allo-HSCT (adults)	25-46 (37)	*Staphylococcus, Corynebacterium, Enterococcus faecalis*	Dental biofilm	Before the conditioning regimen, at aplasia and at engraftment	Higher risk of acute GVHD
				*Vellionella*	Dental biofilm	Before the conditioning regimen, at aplasia and at engraftment	Lower risk of acute GVHD
Campos de Molla et al.	2021	Allo-HSCT (adults)	25-46 (37)	*Solobacterium* absence at preconditioning	Tongue	Before the conditioning regimen and the oral medicine specialist intervention, at aplasia, and at engraftment.	Higher risk of relapse
Ingham et al. (138)	2021	Allo-HSCT (pediatrics)	10-32 (21.4, average)	*Actinomycetaceae*, *Prevotellaceae*, and *Propionibacteriaceae* at preconditioning	Buccal mucosa	At 10 time points over a 1-year period: twice prior to HSCT, on the day of HSCT, weekly during the first month after HSCT, and at three follow-up time points up to 12 months post HSCT.	Higher risk of acute severe GVHD

Relative increase in oral bacteria during HSCT in relation to the outcome and complications. GVHD, graft-versus-host-disease; HSCT, hematopoietic stem cell transplantation; Allo, allogeneic.

## Conclusion

The relationship between oral microbiota and post-transplant complications and survival and recurrence rates has been reviewed. Conditioning regimens, antibiotic combination therapy, and periodontal disease may cause oral dysbiosis, which may lead to direct transfer of pathogenic bacteria to the intestinal tract. Furthermore, local oral inflammation caused by oral dysbiosis may spread to systemic inflammation including GVHD. Further accumulation of evidence is important to determine the mechanism.

## Author contributions

AY, YK and HF conceived of the concept and important topics to include in the article. All authors contributed to writing this review and critical appraisal and review of the final version.

## References

[1] Barnes DW Loutit JF. Treatment of murine leukaemia with X-rays and homologous bone marrow. Ii. Br J Haematol (1957) 3(3):241–52. doi: 10.1111/j.1365-2141.1957.tb05793.x 13460193

[2] Martin PJ Schoch G Fisher L Byers V Anasetti C Appelbaum FR. A retrospective analysis of therapy for acute graft-Versus-Host disease: Initial treatment. Blood (1990) 76(8):1464–72. doi: 10.1182/blood.V76.8.1464.1464 2207321

[3] Ghimire S Weber D Mavin E Wang XN Dickinson AM Holler E. Pathophysiology of gvhd and other hsct-related major complications. Front Immunol (2017) 8:79. doi: 10.3389/fimmu.2017.00079 28373870 PMC5357769

[4] Toubai T Magenau J. Immunopathology and biology-based treatment of steroid-refractory graft-Versus-Host disease. Blood (2020) 136(4):429–40. doi: 10.1182/blood.2019000953 PMC7378454 32526035

[5] Hill GR Crawford JM Cooke KR Brinson YS Pan L Ferrara JLM. Total body irradiation and acute graft-Versus-Host disease: The role of gastrointestinal damage and inflammatory cytokines. Blood (1997) 90(8):3204–13. doi: 10.1182/blood.V90.8.3204 9376604

[6] Zeiser R Blazar BR. Acute graft-Versus-Host disease — biologic process, prevention, and therapy. New Engl J Med (2017) 377(22):2167–79. doi: 10.1056/NEJMra1609337 PMC6034180 29171820

[7] Piñana JL Valcárcel D Fernández-Avilés F Martino R Rovira M Barba P. Mtx or mycophenolate mofetil with csa as gvhd prophylaxis after reduced-intensity conditioning pbsct from hla-identical siblings. Bone Marrow Transplant (2010) 45(9):1449–56. doi: 10.1038/bmt.2009.362 20140024

[8] Bacigalupo A Lamparelli T Bruzzi P Guidi S Alessandrino PE di Bartolomeo P. Antithymocyte globulin for graft-Versus-Host disease prophylaxis in transplants from unrelated donors: 2 randomized studies from gruppo italiano trapianti midollo osseo (Gitmo). Blood (2001) 98(10):2942–7. doi: 10.1182/blood.V98.10.2942 11698275

[9] Mohty M. Mechanisms of action of antithymocyte globulin: T-cell depletion and beyond. Leukemia (2007) 21(7):1387–94. doi: 10.1038/sj.leu.2404683 17410187

[10] Ferrara JLM Levine JE Reddy P Holler E. Graft-Versus-Host disease. Lancet (2009) 373(9674):1550–61. doi: 10.1016/S0140-6736(09)60237-3 PMC2735047 19282026

[11] Zeiser R Socie G Blazar BR. Pathogenesis of acute graft-Versus-Host disease: From intestinal microbiota alterations to donor T cell activation. Br J Haematol (2016) 175(2):191–207. doi: 10.1111/bjh.14295 27619472

[12] Fujiwara H. Crosstalk between intestinal microbiota derived metabolites and tissues in allogeneic hematopoietic cell transplantation. Front Immunol (2021) 12:703298. doi: 10.3389/fimmu.2021.703298 34512627 PMC8429959

[13] Fujiwara H Docampo MD Riwes M Peltier D Toubai T Henig I. Microbial metabolite sensor Gpr43 controls severity of experimental gvhd. Nat Commun (2018) 9(1):3674. doi: 10.1038/s41467-018-06048-w 30201970 PMC6131147

[14] Antin JH Ferrara JL. Cytokine dysregulation and acute graft-Versus-Host disease. Blood (1992) 80(12):2964–8. doi: 10.1182/blood.V80.12.2964.2964 1467511

[15] Malard F Gasc C Plantamura E Dore J. High gastrointestinal microbial diversity and clinical outcome in graft-Versus-Host disease patients. Bone Marrow Transplant (2018) 53(12):1493–7. doi: 10.1038/s41409-018-0254-x PMC6281565 29904128

[16] Qin J Li R Raes J Arumugam M Burgdorf KS Manichanh C. A human gut microbial gene catalogue established by metagenomic sequencing. Nature (2010) 464(7285):59–65. doi: 10.1038/nature08821 20203603 PMC3779803

[17] Espey MG. Role of oxygen gradients in shaping redox relationships between the human intestine and its microbiota. Free Radic Biol Med (2013) 55:130–40. doi: 10.1016/j.freeradbiomed.2012.10.554 23127782

[18] Barker N van de Wetering M Clevers H. The intestinal stem cell. Genes Dev (2008) 22(14):1856–64. doi: 10.1101/gad.1674008 PMC2735277 18628392

[19] Donohoe DR Garge N Zhang X Sun W O'Connell TM Bunger MK. The microbiome and butyrate regulate energy metabolism and autophagy in the mammalian colon. Cell Metab (2011) 13(5):517–26. doi: 10.1016/j.cmet.2011.02.018 PMC3099420 21531334

[20] Litvak Y Byndloss MX Tsolis RM Bäumler AJ. Dysbiotic proteobacteria expansion: A microbial signature of epithelial dysfunction. Curr Opin Microbiol (2017) 39:1–6. doi: 10.1016/j.mib.2017.07.003 28783509

[21] Kelly CJ Zheng L Campbell EL Saeedi B Scholz CC Bayless AJ. Crosstalk between microbiota-derived short-chain fatty acids and intestinal epithelial hif augments tissue barrier function. Cell Host Microbe (2015) 17(5):662–71. doi: 10.1016/j.chom.2015.03.005 PMC4433427 25865369

[22] Peled JU Gomes ALC Devlin SM Littmann ER Taur Y Sung AD. Microbiota as predictor of mortality in allogeneic hematopoietic-cell transplantation. N Engl J Med (2020) 382(9):822–34. doi: 10.1056/NEJMoa1900623 PMC7534690 32101664

[23] Taur Y Jenq RR Perales MA Littmann ER Morjaria S Ling L. The effects of intestinal tract bacterial diversity on mortality following allogeneic hematopoietic stem cell transplantation. Blood (2014) 124(7):1174–82. doi: 10.1182/blood-2014-02-554725 PMC4133489 24939656

[24] Maier L Pruteanu M Kuhn M Zeller G Telzerow A Anderson EE. Extensive impact of non-antibiotic drugs on human gut bacteria. Nature (2018) 555(7698):623–8. doi: 10.1038/nature25979 PMC6108420 29555994

[25] Morjaria S Schluter J Taylor BP Littmann ER Carter RA Fontana E. Antibiotic-induced shifts in fecal microbiota density and composition during hematopoietic stem cell transplantation. Infect Immun (2019) 87(9). doi: 10.1128/IAI.00206-19 PMC6704593 31262981

[26] Kusakabe S Fukushima K Yokota T Hino A Fujita J Motooka D. Enterococcus: A predictor of ravaged microbiota and poor prognosis after allogeneic hematopoietic stem cell transplantation. Biol Blood Marrow Transplant (2020) 26(5):1028–33. doi: 10.1016/j.bbmt.2020.01.019 32018061

[27] Peled JU Devlin SM Staffas A Lumish M Khanin R Littmann ER. Intestinal microbiota and relapse after hematopoietic-cell transplantation. J Clin Oncol (2017) 35(15):1650–9. doi: 10.1200/JCO.2016.70.3348 PMC5455763 28296584

[28] Stein-Thoeringer CK Nichols KB Lazrak A Docampo MD Slingerland AE Slingerland JB. Lactose drives enterococcus expansion to promote graft-Versus-Host disease. Science (2019) 366(6469):1143–9. doi: 10.1126/science.aax3760 PMC7003985 31780560

[29] Beckman MF Morton DS Bahrani Mougeot F Mougeot JC. Allogenic stem cell transplant-associated acute graft versus host disease: A computational drug discovery text mining approach using oral and gut microbiome signatures. Support Care Cancer (2021) 29(4):1765–79. doi: 10.1007/s00520-020-05821-2 33094358

[30] Freifeld AG Bow EJ Sepkowitz KA Boeckh MJ Ito JI Mullen CA. Clinical practice guideline for the use of antimicrobial agents in neutropenic patients with cancer: 2010 update by the infectious diseases society of America. Clin Infect Dis (2011) 52(4):e56–93. doi: 10.1093/cid/cir073 21258094

[31] Shono Y Docampo MD Peled JU Perobelli SM Velardi E Tsai JJ. Increased gvhd-related mortality with broad-spectrum antibiotic use after allogeneic hematopoietic stem cell transplantation in human patients and mice. Sci Transl Med (2016) 8(339):339ra71. doi: 10.1126/scitranslmed.aaf2311 PMC4991773 27194729

[32] Routy B Letendre C Enot D Chénard-Poirier M Mehraj V Séguin NC. The influence of gut-decontamination prophylactic antibiotics on acute graft-Versus-Host disease and survival following allogeneic hematopoietic stem cell transplantation. Oncoimmunology (2017) 6(1):e1258506. doi: 10.1080/2162402x.2016.1258506 28197380 PMC5283637

[33] Weber D Jenq RR Peled JU Taur Y Hiergeist A Koestler J. Microbiota disruption induced by early use of broad-spectrum antibiotics is an independent risk factor of outcome after allogeneic stem cell transplantation. Biol Blood Marrow Transplant (2017) 23(5):845–52. doi: 10.1016/j.bbmt.2017.02.006 PMC5546237 28232086

[34] Khuat LT Le CT Pai CS Shields-Cutler RR Holtan SG Rashidi A. Obesity induces gut microbiota alterations and augments acute graft-Versus-Host disease after allogeneic stem cell transplantation. Sci Transl Med (2020) 12(571). doi: 10.1126/scitranslmed.aay7713 PMC8525601 33239390

[35] Markey KA Schluter J Gomes ALC Littmann ER Pickard AJ Taylor BP. The microbe-derived short-chain fatty acids butyrate and propionate are associated with protection from chronic gvhd. Blood (2020) 136(1):130–6. doi: 10.1182/blood.2019003369 PMC7332893 32430495

[36] Furusawa Y Obata Y Fukuda S Endo TA Nakato G Takahashi D. Commensal microbe-derived butyrate induces the differentiation of colonic regulatory T cells. Nature (2013) 504(7480):446–50. doi: 10.1038/nature12721 24226770

[37] Hamer HM Jonkers D Venema K Vanhoutvin S Troost FJ Brummer RJ. Review article: The role of butyrate on colonic function. Aliment Pharmacol Ther (2008) 27(2):104–19. doi: 10.1111/j.1365-2036.2007.03562.x 17973645

[38] Fujiwara H Seike K Brooks MD Mathew AV Kovalenko I Pal A. Mitochondrial complex ii in intestinal epithelial cells regulates T cell-mediated immunopathology. Nat Immunol (2021) 22(11):1440–51. doi: 10.1038/s41590-021-01048-3 PMC9351914 34686860

[39] Zaura E Brandt BW Teixeira de Mattos MJ Buijs MJ Caspers MP Rashid MU. Same exposure but two radically different responses to antibiotics: Resilience of the salivary microbiome versus long-term microbial shifts in feces. mBio (2015) 6(6):e01693–15. doi: 10.1128/mBio.01693-15 PMC4659469 26556275

[40] Mathewson ND Jenq R Mathew AV Koenigsknecht M Hanash A Toubai T. Gut microbiome-derived metabolites modulate intestinal epithelial cell damage and mitigate graft-Versus-Host disease. Nat Immunol (2016) 17(5):505–13. doi: 10.1038/ni.3400 PMC4836986 26998764

[41] Shono Y van den Brink MRM. Gut microbiota injury in allogeneic haematopoietic stem cell transplantation. Nat Rev Cancer (2018) 18(5):283–95. doi: 10.1038/nrc.2018.10 PMC7485905 29449660

[42] Lu Y Meng R Wang X Xu Y Tang Y Wu J. Caspase-11 signaling enhances graft-Versus-Host disease. Nat Commun (2019) 10(1):4044. doi: 10.1038/s41467-019-11895-2 31492850 PMC6731232

[43] Skaarud KJ Hov JR Hansen SH Kummen M Valeur J Seljeflot I. Mortality and microbial diversity after allogeneic hematopoietic stem cell transplantation: Secondary analysis of a randomized nutritional intervention trial. Sci Rep (2021) 11(1):11593. doi: 10.1038/s41598-021-90976-z 34078971 PMC8172574

[44] Hayase E Hayase T Jamal MA Miyama T Chang CC Ortega MR. Mucus-degrading bacteroides link carbapenems to aggravated graft-Versus-Host disease. Cell (2022) 185(20):3705–19 e14. doi: 10.1016/j.cell.2022.09.007 36179667 PMC9542352

[45] Qiao X Biliński J Wang L Yang T Luo R Fu Y. Safety and efficacy of fecal microbiota transplantation in the treatment of graft-Versus-Host disease. Bone Marrow Transplant (2022) 58:10–19. doi: 10.1038/s41409-022-01824-1 36167905

[46] Atarashi K Suda W Luo C Kawaguchi T Motoo I Narushima S. Ectopic colonization of oral bacteria in the intestine drives T(H)1 cell induction and inflammation. Science (2017) 358(6361):359–65. doi: 10.1126/science.aan4526 PMC5682622 29051379

[47] Li B Ge Y Cheng L Zeng B Yu J Peng X. Oral bacteria colonize and compete with gut microbiota in gnotobiotic mice. Int J Oral Sci (2019) 11(1):10. doi: 10.1038/s41368-018-0043-9 30833566 PMC6399334

[48] Miller WD. The human mouth as a focus of infection. Lancet (1891) 138(3546):340–2. doi: 10.1016/S0140-6736(02)01387-9

[49] Aas JA Paster BJ Stokes LN Olsen I Dewhirst FE. Defining the normal bacterial flora of the oral cavity. J Clin Microbiol (2005) 43(11):5721–32. doi: 10.1128/JCM.43.11.5721-5732.2005 PMC1287824 16272510

[50] Schmidt TS Hayward MR Coelho LP Li SS Costea PI Voigt AY. Extensive transmission of microbes along the gastrointestinal tract. Elife (2019) 8:e42693. doi: 10.7554/eLife.42693 30747106 PMC6424576

[51] Kitamoto S Nagao-Kitamoto H Jiao Y Gillilland MG 3rd Hayashi A Imai J. The intermucosal connection between the mouth and gut in commensal pathobiont-driven colitis. Cell (2020) 182(2):447–62 e14. doi: 10.1016/j.cell.2020.05.048 32758418 PMC7414097

[52] Lloyd-Price J Mahurkar A Rahnavard G Crabtree J Orvis J Hall AB. Strains, functions and dynamics in the expanded human microbiome project. Nature (2017) 550(7674):61–6. doi: 10.1038/nature23889 PMC5831082 28953883

[53] Deo PN Deshmukh R. Oral microbiome: Unveiling the fundamentals. J Oral Maxillofac Pathol (2019) 23(1):122–8. doi: 10.4103/jomfp.JOMFP_304_18 PMC6503789 31110428

[54] Aagaard K Ma J Antony KM Ganu R Petrosino J Versalovic J. The placenta harbors a unique microbiome. Sci Transl Med (2014) 6(237):237ra65. doi: 10.1126/scitranslmed.3008599 PMC4929217 24848255

[55] Zaura E Nicu EA Krom BP Keijser BJF. Acquiring and maintaining a normal oral microbiome: Current perspective. Front Cell Infection Microbiol (2014) 4:85. doi: 10.3389/fcimb.2014.00085 PMC4071637 25019064

[56] Lif Holgerson P Harnevik L Hernell O Tanner AC Johansson I. Mode of birth delivery affects oral microbiota in infants. J Dent Res (2011) 90(10):1183–8. doi: 10.1177/0022034511418973 PMC3173012 21828355

[57] Li Y Caufield PW Dasanayake AP Wiener HW Vermund SH. Mode of delivery and other maternal factors influence the acquisition of streptococcus mutans in infants. J Dent Res (2005) 84(9):806–11. doi: 10.1177/154405910508400905 16109988

[58] Rasiah IA Wong L Anderson SA Sissons CH. Variation in bacterial dgge patterns from human saliva: Over time, between individuals and in corresponding dental plaque microcosms. Arch Oral Biol (2005) 50(9):779–87. doi: 10.1016/j.archoralbio.2005.02.001 15970209

[59] Dewhirst FE Chen T Izard J Paster BJ Tanner AC Yu WH. The human oral microbiome. J Bacteriol (2010) 192(19):5002–17. doi: 10.1128/JB.00542-10 PMC2944498 20656903

[60] Facklam R. What happened to the streptococci: Overview of taxonomic and nomenclature changes. Clin Microbiol Rev (2002) 15(4):613–30. doi: 10.1128/CMR.15.4.613-630.2002 PMC126867 12364372

[61] Lemos JA Palmer SR Zeng L Wen ZT Kajfasz JK Freires IA. The biology of streptococcus mutans. Microbiol Spectr (2019) 7(1). doi: 10.1128/microbiolspec.GPP3-0051-2018 PMC6615571 30657107

[62] Lamont RJ Koo H Hajishengallis G. The oral microbiota: Dynamic communities and host interactions. Nat Rev Microbiol (2018) 16(12):745–59. doi: 10.1038/s41579-018-0089-x PMC6278837 30301974

[63] Mager DL Ximenez-Fyvie LA Haffajee AD Socransky SS. Distribution of selected bacterial species on intraoral surfaces. J Clin Periodontol (2003) 30(7):644–54. doi: 10.1034/j.1600-051x.2003.00376.x 12834503

[64] Segata N Haake SK Mannon P Lemon KP Waldron L Gevers D. Composition of the adult digestive tract bacterial microbiome based on seven mouth surfaces, tonsils, throat and stool samples. Genome Biol (2012) 13(6):R42. doi: 10.1186/gb-2012-13-6-r42 22698087 PMC3446314

[65] Radaic A Kapila YL. The oralome and its dysbiosis: New insights into oral microbiome-host interactions. Comput Struct Biotechnol J (2021) 19:1335–60. doi: 10.1016/j.csbj.2021.02.010 PMC7960681 33777334

[66] Costerton JW Geesey GG Cheng KJ. How bacteria stick. Sci Am (1978) 238(1):86–95. doi: 10.1038/scientificamerican0178-86 635520

[67] Nadell CD Drescher K Wingreen NS Bassler BL. Extracellular matrix structure governs invasion resistance in bacterial biofilms. ISME J (2015) 9(8):1700–9. doi: 10.1038/ismej.2014.246 PMC4511925 25603396

[68] Baker JL Bor B Agnello M Shi W He X. Ecology of the oral microbiome: Beyond bacteria. Trends Microbiol (2017) 25(5):362–74. doi: 10.1016/j.tim.2016.12.012 PMC5687246 28089325

[69] Peters BA Wu J Hayes RB Ahn J. The oral fungal mycobiome: Characteristics and relation to periodontitis in a pilot study. BMC Microbiol (2017) 17(1):157. doi: 10.1186/s12866-017-1064-9 28701186 PMC5508751

[70] Sztukowska MN Dutton LC Delaney C Ramsdale M Ramage G Jenkinson HF. Community development between porphyromonas gingivalis and candida albicans mediated by inlj and Als3. mBio (2018) 9(2). doi: 10.1128/mBio.00202-18 PMC5915736 29691333

[71] Dahlen G. Bacterial infections of the oral mucosa. Periodontol 2000 (2009) 49:13–38. doi: 10.1111/j.1600-0757.2008.00295.x 19152524

[72] Marsh PD Do T Beighton D Devine DA. Influence of saliva on the oral microbiota. Periodontol 2000 (2016) 70(1):80–92. doi: 10.1111/prd.12098 26662484

[73] Zalewska A Knaś M Kuźmiuk A Waszkiewicz N Niczyporuk M Waszkiel D. Salivary innate defense system in type 1 diabetes mellitus in children with mixed and permanent dentition. Acta Odontol Scand (2013) 71(6):1493–500. doi: 10.3109/00016357.2013.773071 23445270

[74] Petersen C Round JL. Defining dysbiosis and its influence on host immunity and disease. Cell Microbiol (2014) 16(7):1024–33. doi: 10.1111/cmi.12308 PMC4143175 24798552

[75] Santonocito S Giudice A Polizzi A Troiano G Merlo EM Sclafani R. A cross-talk between diet and the oral microbiome: Balance of nutrition on inflammation and immune system's response during periodontitis. Nutrients (2022) 14(12). doi: 10.3390/nu14122426 PMC9227938 35745156

[76] Wade WG. Resilience of the oral microbiome. Periodontol 2000 (2021) 86(1):113–22. doi: 10.1111/prd.12365 33690989

[77] Shaalan A Lee S Feart C Garcia-Esquinas E Gomez-Cabrero D Lopez-Garcia E. Alterations in the oral microbiome associated with diabetes, overweight, and dietary components. Front Nutr (2022) 9:914715. doi: 10.3389/fnut.2022.914715 35873415 PMC9298547

[78] Jia YJ Liao Y He YQ Zheng MQ Tong XT Xue WQ. Association between oral microbiota and cigarette smoking in the Chinese population. Front Cell Infect Microbiol (2021) 11:658203. doi: 10.3389/fcimb.2021.658203 34123872 PMC8195269

[79] Kouidhi B Al Qurashi YM Chaieb K. Drug resistance of bacterial dental biofilm and the potential use of natural compounds as alternative for prevention and treatment. Microb Pathog (2015) 80:39–49. doi: 10.1016/j.micpath.2015.02.007 25708507

[80] Tuganbaev T Yoshida K Honda K. The effects of oral microbiota on health. Science (2022) 376(6596):934–6. doi: 10.1126/science.abn1890 35617380

[81] Kinane DF Stathopoulou PG Papapanou PN. Periodontal diseases. Nat Rev Dis Primers (2017) 3(1):17038. doi: 10.1038/nrdp.2017.38 28805207

[82] Eke PI Wei L Thornton-Evans GO Borrell LN Borgnakke WS Dye B. Risk indicators for periodontitis in us adults: Nhanes 2009 to 2012. J Periodontol (2016) 87(10):1174–85. doi: 10.1902/jop.2016.160013 PMC11370315 27367420

[83] Hajishengallis G Maekawa T Abe T Hajishengallis E Lambris JD. Complement involvement in periodontitis: Molecular mechanisms and rational therapeutic approaches. Adv Exp Med Biol (2015) 865:57–74. doi: 10.1007/978-3-319-18603-0_4 26306443 PMC4562417

[84] Zhang Z Liu S Zhang S Li Y Shi X Liu D. Porphyromonas gingivalis outer membrane vesicles inhibit the invasion of fusobacterium nucleatum into oral epithelial cells by downregulating fada and foma. J Periodontol (2021) 93(4):515–25. doi: 10.1002/jper.21-0144 PMC9415117 34458990

[85] Groeger S Meyle J. Oral mucosal epithelial cells. Front Immunol (2019) 10:208. doi: 10.3389/fimmu.2019.00208 30837987 PMC6383680

[86] de Molon RS de Avila ED Boas Nogueira AV Chaves de Souza JA Avila-Campos MJ de Andrade CR. Evaluation of the host response in various models of induced periodontal disease in mice. J Periodontol (2014) 85(3):465–77. doi: 10.1902/jop.2013.130225 23805811

[87] Izard J Sasaki H Kent R. Pathogenicity of treponema denticola wild-type and mutant strain tested by an active mode of periodontal infection using microinjection. Int J Dent (2012) 2012:549169. doi: 10.1155/2012/549169 22829826 PMC3398590

[88] Baker PJ Dixon M Evans RT Roopenian DC. Heterogeneity of porphyromonas gingivalis strains in the induction of alveolar bone loss in mice. Oral Microbiol Immunol (2000) 15(1):27–32. doi: 10.1034/j.1399-302x.2000.150105.x 11155161

[89] Zhang Z Liu S Zhang S Li Y Shi X Liu D. Porphyromonas gingivalis outer membrane vesicles inhibit the invasion of fusobacterium nucleatum into oral epithelial cells by downregulating fada and foma. J Periodontol (2022) 93(4):515–25. doi: 10.1002/jper.21-0144 PMC9415117 34458990

[90] Soffritti I D'Accolti M Fabbri C Passaro A Manfredini R Zuliani G. Oral microbiome dysbiosis is associated with symptoms severity and local Immune/Inflammatory response in covid-19 patients: A cross-sectional study. Front Microbiol (2021) 12:687513. doi: 10.3389/fmicb.2021.687513 34248910 PMC8261071

[91] Rafiqul Islam SM Foysal MJ Hoque MN Mehedi HMH Rob MA Salauddin A. Dysbiosis of oral and gut microbiomes in sars-Cov-2 infected patients in Bangladesh: Elucidating the role of opportunistic gut microbes. Front Med (Lausanne) (2022) 9:821777. doi: 10.3389/fmed.2022.821777 35237631 PMC8882723

[92] Zuza EC Pires JR de Almeida AA Toledo BEC Guimaraes-Stabili MR Junior CR. Evaluation of recurrence of periodontal disease after treatment in obese and normal weight patients: Two-year follow-up. J Periodontol (2020) 91(9):1123–31. doi: 10.1002/JPER.19-0534 32010963

[93] Billings F. Chronic focal infections and their etiologic relations to arthritis and nephritis. Arch Internal Med (1912) IX(4):484–98. doi: 10.1001/archinte.1912.00060160087007

[94] Grigg JB Sonnenberg GF. Host-microbiota interactions shape local and systemic inflammatory diseases. J Immunol (2017) 198(2):564–71. doi: 10.4049/jimmunol.1601621 PMC5228396 28069751

[95] Peng X Cheng L You Y Tang C Ren B Li Y. Oral microbiota in human systematic diseases. Int J Oral Sci (2022) 14(1):14. doi: 10.1038/s41368-022-00163-7 35236828 PMC8891310

[96] Kim SS Ruiz VE Carroll JD Moss SF. Helicobacter pylori in the pathogenesis of gastric cancer and gastric lymphoma. Cancer Lett (2011) 305(2):228–38. doi: 10.1016/j.canlet.2010.07.014 PMC2980557 20692762

[97] Whitmore SE Lamont RJ. Oral bacteria and cancer. PloS Pathog (2014) 10(3):e1003933. doi: 10.1371/journal.ppat.1003933 24676390 PMC3968118

[98] Nagy KN Sonkodi I Szöke I Nagy E Newman HN. The microflora associated with human oral carcinomas. Oral Oncol (1998) 34(4):304–8. doi: 10.1016/S1368-8375(98)80012-2 9813727

[99] Katz J Onate MD Pauley KM Bhattacharyya I Cha S. Presence of porphyromonas gingivalis in gingival squamous cell carcinoma. Int J Oral Sci (2011) 3(4):209–15. doi: 10.4248/ijos11075 PMC3469978 22010579

[100] Ahn J Segers S Hayes RB. Periodontal disease, porphyromonas gingivalis serum antibody levels and orodigestive cancer mortality. Carcinogenesis (2012) 33(5):1055–8. doi: 10.1093/carcin/bgs112 PMC3334514 22367402

[101] Mao S Park Y Hasegawa Y Tribble GD James CE Handfield M. Intrinsic apoptotic pathways of gingival epithelial cells modulated by porphyromonas gingivalis. Cell Microbiol (2007) 9(8):1997–2007. doi: 10.1111/j.1462-5822.2007.00931.x 17419719 PMC2886729

[102] Kuboniwa M Hasegawa Y Mao S Shizukuishi S Amano A Lamont RJ. P. gingivalis accelerates gingival epithelial cell progression through the cell cycle. Microbes Infect (2008) 10(2):122–8. doi: 10.1016/j.micinf.2007.10.011 PMC2311419 18280195

[103] McCoy AN Araújo-Pérez F Azcárate-Peril A Yeh JJ Sandler RS Keku TO. Fusobacterium is associated with colorectal adenomas. PloS One (2013) 8(1):e53653. doi: 10.1371/journal.pone.0053653 23335968 PMC3546075

[104] Sun CH Li BB Wang B Zhao J Zhang XY Li TT. The role of fusobacterium nucleatum in colorectal cancer: From carcinogenesis to clinical management. Chronic Dis Transl Med (2019) 5(3):178–87. doi: 10.1016/j.cdtm.2019.09.001 PMC6926109 31891129

[105] Dong J Li Y Xiao H Zhang S Wang B Wang H. Oral microbiota affects the efficacy and prognosis of radiotherapy for colorectal cancer in mouse models. Cell Rep (2021) 37(4):109886. doi: 10.1016/j.celrep.2021.109886 34706245

[106] Wang Y Xue J Zhou X You M Du Q Yang X. Oral microbiota distinguishes acute lymphoblastic leukemia pediatric hosts from healthy populations. PloS One (2014) 9(7):e102116. doi: 10.1371/journal.pone.0102116 25025462 PMC4099009

[107] Genco RJ Graziani F Hasturk H. Effects of periodontal disease on glycemic control, complications, and incidence of diabetes mellitus. Periodontol 2000 (2020) 83(1):59–65. doi: 10.1111/prd.12271 32385875

[108] Tong Y Zheng L Qing P Zhao H Li Y Su L. Oral microbiota perturbations are linked to high risk for rheumatoid arthritis. Front Cell Infect Microbiol (2019) 9:475. doi: 10.3389/fcimb.2019.00475 32039051 PMC6987375

[109] Gao L Cheng Z Zhu F Bi C Shi Q Chen X. The oral microbiome and its role in systemic autoimmune diseases: A systematic review of big data analysis. Front Big Data (2022) 5:927520. doi: 10.3389/fdata.2022.927520 35844967 PMC9277227

[110] DeStefano F Anda RF Kahn HS Williamson DF Russell CM. Dental disease and risk of coronary heart disease and mortality. Bmj (1993) 306(6879):688–91. doi: 10.1136/bmj.306.6879.688 PMC1677081 8471920

[111] Chhibber-Goel J Singhal V Bhowmik D Vivek R Parakh N Bhargava B. Linkages between oral commensal bacteria and atherosclerotic plaques in coronary artery disease patients. NPJ Biofilms Microbiomes (2016) 2:7. doi: 10.1038/s41522-016-0009-7 28649401 PMC5460270

[112] Kamer AR Craig RG Niederman R Fortea J de Leon MJ. Periodontal disease as a possible cause for alzheimer's disease. Periodontol 2000 (2020) 83(1):242–71. doi: 10.1111/prd.12327 32385876

[113] Werber T Bata Z Vaszine ES Berente DB Kamondi A Horvath AA. The association of periodontitis and alzheimer's disease: How to hit two birds with one stone. J Alzheimers Dis (2021) 84(1):1–21. doi: 10.3233/jad-210491 34511500

[114] Paganini-Hill A White SC Atchison KA. Dentition, dental health habits, and dementia: The leisure world cohort study. J Am Geriatr Soc (2012) 60(8):1556–63. doi: 10.1111/j.1532-5415.2012.04064.x 22860988

[115] Khasnobish A Takayasu L Watanabe KI Nguyen TTT Arakawa K Hotta O. Dysbiosis in the salivary microbiome associated with iga nephropathy-a Japanese cohort study. Microbes Environ (2021) 36(2). doi: 10.1264/jsme2.ME21006 PMC8209455 34078780

[116] Fardini Y Chung P Dumm R Joshi N Han YW. Transmission of diverse oral bacteria to murine placenta: Evidence for the oral microbiome as a potential source of intrauterine infection. Infect Immun (2010) 78(4):1789–96. doi: 10.1128/IAI.01395-09 PMC2849412 20123706

[117] Khadija B Badshah L Siddiqa A Rehman B Anjum S Saeed A. Dysbiosis in salivary bacterial diversity of postpartum females and its association with oral health problems and apos. Curr Res Microb Sci (2021) 2:100032. doi: 10.1016/j.crmicr.2021.100032 34841323 PMC8610344

[118] Read E Curtis MA Neves JF. The role of oral bacteria in inflammatory bowel disease. Nat Rev Gastroenterol Hepatol (2021) 18(10):731–42. doi: 10.1038/s41575-021-00488-4 34400822

[119] Martinsen TC Bergh K Waldum HL. Gastric juice: A barrier against infectious diseases. Basic Clin Pharmacol Toxicol (2005) 96(2):94–102. doi: 10.1111/j.1742-7843.2005.pto960202.x 15679471

[120] Collins SL Stine JG Bisanz JE Okafor CD Patterson AD. Bile acids and the gut microbiota: Metabolic interactions and impacts on disease. Nat Rev Microbiol (2022). doi: 10.1038/s41579-022-00805-x PMC12536349 36253479

[121] Bao J Li L Zhang Y Wang M Chen F Ge S. Periodontitis may induce gut microbiota dysbiosis *Via* salivary microbiota. Int J Oral Sci (2022) 14(1):32. doi: 10.1038/s41368-022-00183-3 35732628 PMC9217941

[122] Kato T Yamazaki K Nakajima M Date Y Kikuchi J Hase K. Oral administration of porphyromonas gingivalis alters the gut microbiome and serum metabolome. mSphere (2018) 3(5):e00460–18. doi: 10.1128/mSphere.00460-18 PMC6193602 30333180

[123] Zhang X Zhang D Jia H Feng Q Wang D Liang D. The oral and gut microbiomes are perturbed in rheumatoid arthritis and partly normalized after treatment. Nat Med (2015) 21(8):895–905. doi: 10.1038/nm.3914 26214836

[124] Flynn KJ Baxter NT Schloss PD. Metabolic and community synergy of oral bacteria in colorectal cancer. mSphere (2016) 1(3)e00102–16. doi: 10.1128/mSphere.00102-16 PMC4888883 27303740

[125] Gevers D Kugathasan S Denson LA Vazquez-Baeza Y Van Treuren W Ren B. The treatment-naive microbiome in new-onset crohn's disease. Cell Host Microbe (2014) 15(3):382–92. doi: 10.1016/j.chom.2014.02.005 PMC4059512 24629344

[126] Imhann F Bonder MJ Vich Vila A Fu J Mujagic Z Vork L. Proton pump inhibitors affect the gut microbiome. Gut (2016) 65(5):740–8. doi: 10.1136/gutjnl-2015-310376 PMC4853569 26657899

[127] Hajishengallis G. Periodontitis: From microbial immune subversion to systemic inflammation. Nat Rev Immunol (2015) 15(1):30–44. doi: 10.1038/nri3785 25534621 PMC4276050

[128] Berger K Schopohl D Bollig A Strobach D Rieger C Rublee D. Burden of oral mucositis: A systematic review and implications for future research. Oncol Res Treat (2018) 41(6):399–405. doi: 10.1159/000487085 29734184

[129] Ruescher TJ Sodeifi A Scrivani SJ Kaban LB Sonis ST. The impact of mucositis on alpha-hemolytic streptococcal infection in patients undergoing autologous bone marrow transplantation for hematologic malignancies. Cancer (1998) 82(11):2275–81. doi: 10.1002/(sici)1097-0142(19980601)82:11<2275::Aid-cncr25>3.0.Co;2-q 9610710

[130] Spielberger R Stiff P Bensinger W Gentile T Weisdorf D Kewalramani T. Palifermin for oral mucositis after intensive therapy for hematologic cancers. N Engl J Med (2004) 351(25):2590–8. doi: 10.1056/NEJMoa040125 15602019

[131] Vera-Llonch M Oster G Ford CM Lu J Sonis S. Oral mucositis and outcomes of allogeneic hematopoietic stem-cell transplantation in patients with hematologic malignancies. Support Care Cancer (2007) 15(5):491–6. doi: 10.1007/s00520-006-0176-9 17139495

[132] Shouval R Eshel A Dubovski B Kuperman AA Danylesko I Fein JA. Patterns of salivary microbiota injury and oral mucositis in recipients of allogeneic hematopoietic stem cell transplantation. Blood Adv (2020) 4(13):2912–7. doi: 10.1182/bloodadvances.2020001827 PMC7362373 32598476

[133] Muro M Soga Y Higuchi T Kataoka K Ekuni D Maeda Y. Unusual oral mucosal microbiota after hematopoietic cell transplantation with glycopeptide antibiotics: Potential association with pathophysiology of oral mucositis. Folia Microbiol (Praha) (2018) 63(5):587–97. doi: 10.1007/s12223-018-0596-1 29532421

[134] Laheij A Rozema FR Brennan MT von Bultzingslowen I van Leeuwen SJM Potting C. Long-term analysis of resilience of the oral microbiome in allogeneic stem cell transplant recipients. Microorganisms (2022) 10(4):734. doi: 10.3390/microorganisms10040734 35456787 PMC9030553

[135] Sugita J Matsushita T Kashiwazaki H Kosugi M Takahashi S Wakasa K. Efficacy of folinic acid in preventing oral mucositis in allogeneic hematopoietic stem cell transplant patients receiving mtx as prophylaxis for gvhd. Bone Marrow Transplant (2012) 47(2):258–64. doi: 10.1038/bmt.2011.53 21423118

[136] de Molla VC Heidrich V Bruno JS Knebel FH Miranda-Silva W Asprino PF. Disruption of the oral microbiota is associated with a higher risk of relapse after allogeneic hematopoietic stem cell transplantation. Sci Rep (2021) 11(1):17552. doi: 10.1038/s41598-021-96939-8 34475459 PMC8413296

[137] Heidrich V Bruno JS Knebel FH de Molla VC Miranda-Silva W Asprino PF. Dental biofilm microbiota dysbiosis is associated with the risk of acute graft-Versus-Host disease after allogeneic hematopoietic stem cell transplantation. Front Immunol (2021) 12:692225. doi: 10.3389/fimmu.2021.692225 34220852 PMC8250416

[138] Ingham AC Kielsen K Mordhorst H Ifversen M Aarestrup FM Muller KG. Microbiota long-term dynamics and prediction of acute graft-Versus-Host disease in pediatric allogeneic stem cell transplantation. Microbiome (2021) 9(1):148. doi: 10.1186/s40168-021-01100-2 34183060 PMC8240369

[139] Ames NJ Barb JJ Ranucci A Kim H Mudra SE Cashion AK. The oral microbiome of patients undergoing treatment for severe aplastic anemia: A pilot study. Ann Hematol (2019) 98(6):1351–65. doi: 10.1007/s00277-019-03599-w 30919073

[140] Oku S Takeshita T Futatsuki T Kageyama S Asakawa M Mori Y. Disrupted tongue microbiota and detection of nonindigenous bacteria on the day of allogeneic hematopoietic stem cell transplantation. PloS Pathog (2020) 16(3):e1008348. doi: 10.1371/journal.ppat.1008348 32150591 PMC7082065

[141] Ames NJ Sulima P Ngo T Barb J Munson PJ Paster BJ. A characterization of the oral microbiome in allogeneic stem cell transplant patients. PloS One (2012) 7(10):e47628. doi: 10.1371/journal.pone.0047628 23144704 PMC3483166

